# Successful defibrotide treatment of a patient with veno-occlusive disease after living-donor liver transplantation: A case report

**DOI:** 10.1097/MD.0000000000026463

**Published:** 2021-06-25

**Authors:** Tae Beom Lee, Kwangho Yang, Hyo Jung Ko, Jae Ryong Shim, Byung Hyun Choi, Jung Hee Lee, Je Ho Ryu

**Affiliations:** a Division of Hepato-Biliary-Pancreatic Surgery and Transplantation, Department of Surgery; b Research Institute for Convergence of Biomedical Science and Technology; c Department of Pathology, Pusan National University Yangsan Hospital, 20 Geumo-ro, Mulgeum-eup, Yangsan, Republic of Korea.

**Keywords:** case report, defibrotide, liver transplantation, veno-occlusive disease

## Abstract

**Rationale:**

Veno-occlusive disease (VOD) is characterized by painful hepatomegaly, ascites, weight gain, and jaundice with nonthrombotic, fibrous obliteration of the centrilobular hepatic veins. VOD after liver transplantation is a rare complication, with an incidence of approximately 2%; however, it can be life-threatening in severe cases. The precise etiology and mechanism of VOD after liver transplantation remains unclear. Acute cellular rejection, antibody-mediated rejection, and treatment with tacrolimus or azathioprine may be associated with the development of VOD after liver transplantation. Additionally, the optimal treatment of VOD after liver transplantation has not yet been established and focuses on supportive care. Defibrotide is an anti-ischemic and antithrombotic drug with no systemic anticoagulant effects. Moreover, only a few reports have investigated the use of defibrotide for VOD after liver transplantation, which has shown promising results.

**Patient concerns:**

A 39-year-old woman with primary biliary cholangitis underwent living-donor liver transplantation at our center. She experienced right upper quadrant pain with increased ascites, pleural effusion, and weight gain on postoperative day 14.

**Diagnoses:**

Imaging and pathological tests showed no evidence of rejection or vessel complications. VOD was diagnosed clinically based on the findings of weight gain, ascites, jaundice, and pathological biopsy.

**Interventions:**

Defibrotid, 25 mg/kg/day, was administered intravenously for 21 days.

**Outcomes:**

She showed complete clinical resolution of the VOD.

**Lessons:**

Herein, we report a case of successful defibrotide treatment of VOD after living-donor liver transplantation.

## 1 Introduction

Veno-occlusive disease (VOD), also known as sinusoidal obstruction syndrome, is characterized by hepatomegaly, pain, ascites, weight gain, and jaundice resulting from nonthrombotic, fibrous obliteration, and congestion of the centrilobular hepatic veins.^[1]^ VOD was first reported in patients who had consumed Senecio tea containing pyrrolizidine alkaloids.^[2]^ In the transplantation setting, VOD more commonly develops in patients with hematological disorders who have undergone stem cell transplantation (SCT) preceded by chemoradiation therapy rather than in patients who have undergone solid organ transplantation.^[3]^ For liver transplantation (LT), the reported incidence of VOD is approximately 2%,^[4,5]^ and VOD after LT is thought to be associated with acute cellular rejection (ACR), antibody-mediated rejection (AMR), or use of tacrolimus or azathioprine.^[4,6,7]^ Treatment for VOD after SCT is mainly supportive, and the recovery rate is up to 85% in mild cases.^[8]^ In contrast, the result is far less favorable after LT, with mortality notes in up to 63% of cases.^[4]^ To date, the optimal treatment for VOD after LT is unknown.

Defibrotide has anti-ischemic and antithrombotic properties without significant systemic anticoagulant effects.^[9]^ A study reported that defibrotide resolved VOD in 41% of patients who underwent SCT.^[10]^ Mor et al assessed defibrotide treatment for VOD after LT. In their study, the authors showed a promising outcome, where 1 out of 2 patients survived.^[11]^ Herein, we report the case of a patient with VOD after living-donor liver transplantation (LDLT), who was successfully treated with defibrotide.

## 2 Case report

A 39-year-old woman underwent LDLT for primary biliary cholangitis (PBC) and autoimmune hepatitis. Azathioprine and prednisolone were previously administered to treat PBC and autoimmune hepatitis before LDLT. Owing to the underlying disease, her serum bilirubin level increased to 27 mg/dL, and serum aspartate aminotransferase (AST) and alanine aminotransferase (ALT) levels were 73 IU/L and 41 IU/L, respectively. She received an ABO-compatible, extended right lobe graft from her 51-year-old sister. The patient's preoperative model for end-stage liver disease score was 23 points, and the Child-Pugh score was 11 points with uncontrolled ascites, repeated spontaneous bacterial peritonitis, and esophageal varices.

The middle hepatic vein of the graft was lengthened using a polytetrafluoroethylene graft for convenient anastomosis to the recipient's middle and left hepatic veins. The liver graft had an 8-mm right inferior hepatic vein, which was anastomosed to the recipient's inferior vena cava. In terms of biliary anastomosis, there were 2 openings of hepatic ducts from the cut surface of the liver graft, which were anastomosed using separate hepaticojejunostomies for each hepatic duct opening with respective internal stents. The graft weight was 652 g, and the graft-to-recipient weight ratio was 1.15.

**Figure 1:**
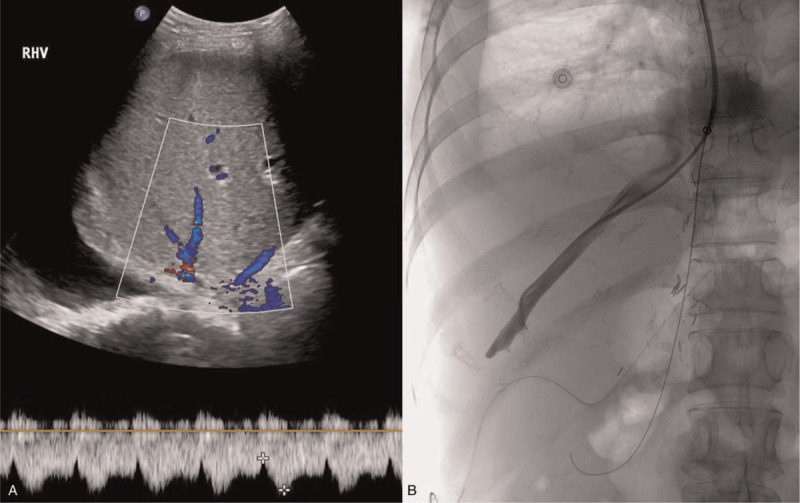
(A) Doppler ultrasound image showing normal hepatic hemodynamics without obstruction of hepatic veins on postoperative day 14. (B) Transjugular hepatic venography on the same day also shows no obstruction or stenosis of the right hepatic vein.

**Figure 2:**
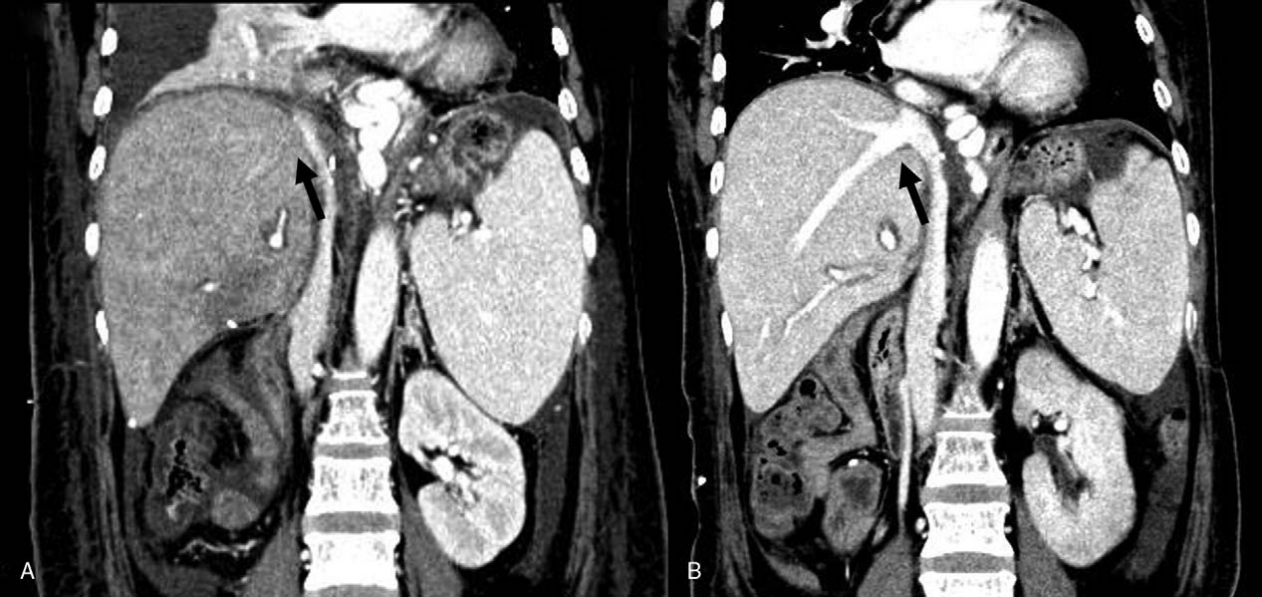
(A) Abdominal computed tomography image on postoperative day 14 showing heterogeneous enhancement of the liver graft with massive pleural effusion and ascites. The right hepatic vein (arrow) is obscured. (B) Abdominal computed tomography on postoperative day 43 showing improvement of the veno-occlusive disease. Pleural effusion and ascites are markedly decreased. The enhanced pattern of the liver graft is homogeneous, and the right hepatic vein (arrow) reappears.

**Figure 3:**
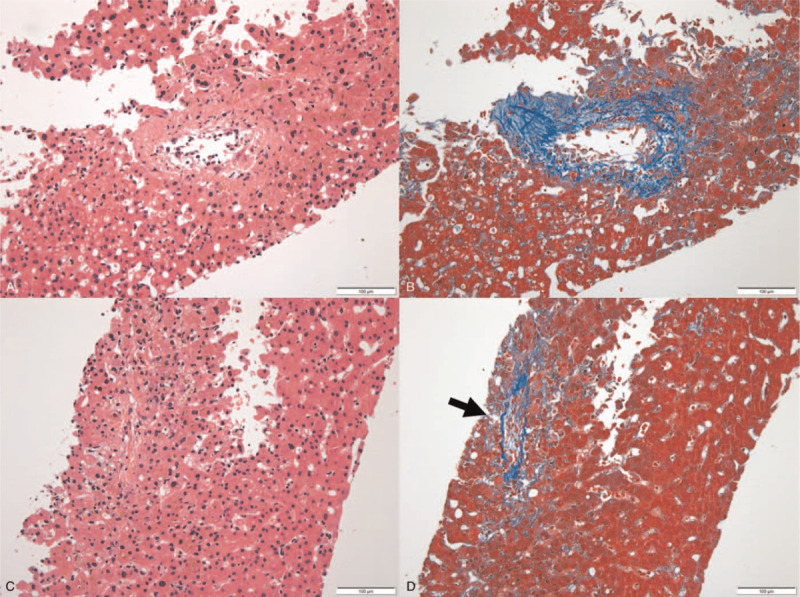
Biopsy specimen showing central venous lesions. The dropout of endothelial cells is associated with inflammation and perivenular fibrosis in a central vein (A and B). In the adjacent parenchyma, central venous obstruction is seen. A vascular structure that cannot be identified in a hematoxylin–eosin stain (C) becomes evident in a Masson trichrome stain (D, arrow). (A and C: hematoxylin–eosin stain; magnification 200×; B and D: Masson trichrome stain; magnification 200×).

Following transplantation, the immunosuppressive regimen included tacrolimus and prednisolone. The patient's postoperative course was uneventful for 4 days. At that point, her serum bilirubin level declined to 7 mg/dL, and her AST and ALT levels were near normal at 38 IU/L and 72 IU/L, respectively. However, from postoperative day (POD) 5, serum bilirubin and hepatic transaminase levels were rapidly increasing: serum bilirubin, 13.9 mg/dL; AST, 152 IU/L; and ALT, 159 IU/L. The hepatic artery, as well as the portal and hepatic veins, were patent on Doppler ultrasonography. Abdominal computed tomography (CT) revealed a decreased size of the hepatic veins with low perivascular density. We provisionally diagnosed ACR on a clinical basis and started methylprednisolone pulse therapy (10 mg/kg for 3 days). Improvement of liver function tests was observed after this therapy: serum bilirubin, 10.6 mg/dL; AST, 85 IU/L; and ALT, 178 IU/L. However, serum bilirubin and hepatic transaminase levels subsequently increased, and the patient experienced right upper quadrant pain with increased ascites, pleural effusion, and weight gain of 6 kg. On POD 14, Doppler ultrasonography revealed normal flow of the hepatic artery and both portal and hepatic veins, without obstruction of the hepatic veins (Fig. 1A); however, abdominal CT revealed a decreased diameter of these veins with low perivascular density, heterogeneous enhancement of the liver graft, and increased amounts of ascites and pleural effusion (Fig. 2A). Therefore, we suspected VOD and simultaneously performed hepatic venography and transjugular liver biopsy. Hepatic venography showed no obstruction or stenosis of the hepatic veins (Fig. 1B). The histological results showed obliterative venulitis with perivenular inflammation, congestion, and hemorrhagic necrosis, and there was no evidence of ACR (Fig. 3).

Clinical and pathological findings suggested VOD. We discontinued tacrolimus administration to avoid its negative effects on VOD and changed our immunosuppressive regimen to a combination of cyclosporine A and prednisolone. After this, the best conservative management for this patient using prostaglandin E1 (PGE1), antithrombin III (ATIII) concentrate, diuretics, and albumin was initiated. However, the patient's body weight or amount of ascites did not reduce.

**Figure 4:**
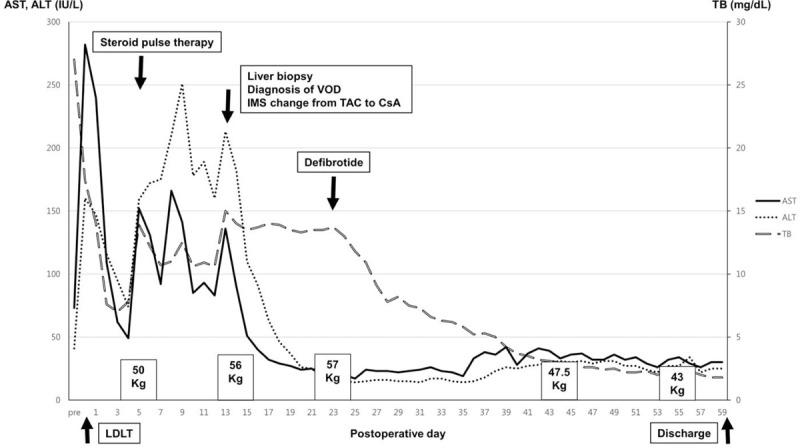
Graph showing the clinical course of the patient after living-donor liver transplantation. After intravenous administration of defibrotide, serum bilirubin and body weight of the patient gradually decreased. ALT = alanine aminotransferase, AST = aspartate aminotransferase, CsA = cyclosporine A, IMS = immunosuppressant, LDLT = living-donor liver transplantation, Pre = preoperative day, TAC = tacrolimus, TB = total bilirubin, VOD = veno-occlusive disease.

On POD 23, intravenous defibrotide (25 mg/kg), divided into 4 doses over 21 days, was administered. During defibrotide administration, the patient's body weight decreased by 9.5 kg compared with the initial body weight (57 kg). Abdominal CT on POD 43 showed an increased size of the hepatic vein diameter and markedly decreased amounts of ascites and pleural effusion (Fig. 2B). Serum bilirubin decreased to 2.7 mg/dL, with AST and ALT being 33 IU/L and 31 IU/L, respectively. The patient was discharged on POD 59, and she is currently alive with normal liver function 25 months after LDLT. No unwanted effects of defibrotide were observed. The clinical course of the patients is summarized in Figure 4.

## 3 Discussion

VOD is a common complication of SCT, but rarely occurs after solid organ transplantation. According to previous studies, VOD occurred after LT in approximately 2% of large study populations.^[4,5]^ The diagnosis of VOD is primarily based on widely used clinical criteria including modified Seattle or Baltimore criteria.^[3]^ Jaundice with serum bilirubin > 2 mg/dL, hepatomegaly, right upper quadrant pain, weight gain more than 2% from the pre-transplant weight, and ascites are noted as diagnostic valuables. Liver biopsy can be helpful for patients in whom the diagnosis of VOD is unclear, and other diagnoses should be excluded.^[3]^

Because the clinical symptoms that accompany VOD are nonspecific, the diagnosis of VOD can be challenging in patients who underwent LT compared with those who underwent other types of transplantation. Jaundice after LT is common and is related to several factors such as biliary anastomosis site complications, acute rejection, recurrence of viral hepatitis, ischemic injury of the graft, and drug toxicity. Ascites after LT can be related to hepatic outflow obstruction, small-for-size syndrome, and viral hepatitis. Thus, it can be difficult to depend on the clinical symptoms to diagnose VOD after LT. Therefore, for the accurate diagnosis of VOD after LT, it is crucial to distinguish VOD from other complications with clinical correlation, including pathologic and imaging findings.

Pathologically, VOD shows nonthrombotic, fibrous obliterative lesions of the hepatic veins by connective tissue and centrilobular necrosis and congestion.^[1]^ Early changes include cell damage to sinusoidal and small hepatic vein endothelium, whereas late changes comprise the fibrous obliteration of small hepatic veins.^[12]^ In the present case, we histologically confirmed central venous lesions with dropout of endothelial cells associated with perivenular fibrosis and obstruction of the hepatic veins.

Imaging studies, including CT, fibroscan, and magnetic resonance imaging can aid in diagnosing VOD after LT. Ascites, pleural effusion, and narrowing of the main hepatic veins are considered the most frequent signs of VOD on CT and magnetic resonance images.^[13]^ In a recent study, the authors suggested that heterogeneous hypoattenuation and patchy liver enhancement in the portal venous or equilibrium phase may be the most important CT features of VOD.^[14]^ In the present study, abdominal CT on POD 14 showed markedly increased pleural effusion and ascites, narrowing of the right hepatic vein, and heterogeneous enhancement of the liver parenchyma. Doppler ultrasonography and hepatic venography were performed for the differential diagnosis of VOD, and there was no evidence of hepatic inflow or outflow obstruction.

According to previous studies, VOD after LT is commonly associated with azathioprine treatment.^[4,6]^ However, azathioprine has no further benefits after LT and has been replaced by other immunosuppressive agents such as tacrolimus or cyclosporine A. Therefore, recent studies have suggested that immunological reactions, including ACR and AMR, play a role in the development of VOD after LT.^[15,16]^ Tacrolimus can induce the dysregulation of endothelial cells and primarily metabolize in the liver; consequently, hepatic dysfunction may affect its metabolism. Thus, it may contribute to the development of VOD.^[17,18]^ Shen et al reported 2 cases of VOD caused by tacrolimus after LT.^[17,19]^ In their studies, 1 recipient recovered following tacrolimus withdrawal; however, another recipient did not recover and eventually underwent re-transplantation. In the present case, the exact cause of VOD remains uncertain, but there are some possibilities related to the development of VOD. Notably, the patient had taken 50 mg azathioprine daily for about 4 months as PBC treatment until just before LDLT. Therefore, azathioprine may have caused the VOD in this patient. Tacrolimus is also possible, even though the serum trough level of tacrolimus in this patient was within the conventional range. In the early period after LDLT, some clinical signs were indicative of ACR, but this patient presented no pathological evidence of ACR. Thus, it is uncertain whether ACR induced VOD in the present case.

Current management of VOD is primarily supportive care with fluid management, adequate oxygenation, and transfusion to minimize ischemic injury of the liver and prevent the release of hepatotoxins.^[20]^ In patients with mild-to-moderate VOD, supportive care including fluid limitation, diuretics, and paracentesis can be effective. By contrast, patients with severe VOD require further treatment beyond supportive care, but therapeutic options for severe VOD remain limited.

Anticoagulation and thrombolytic agents such as tissue plasminogen activator (t-PA), heparin, PGE1, and ATIII concentrate have been used for VOD treatment. Although t-PA with or without heparin showed some improvement in VOD, its application is limited due to the occurrence of fatal bleeding.^[20–22]^ The use of ATIII concentrate and PGE1 showed no beneficial effect on VOD.^[23,24]^ A transjugular intrahepatic portosystemic stent-shunt may support the improvement of ascites; however, it has no benefit for jaundice and patient survival.^[5,25]^ It may play a role as a bridging therapy to re-LT by improving VOD-induced severe portal hypertension.

Defibrotide is a single-stranded polydeoxyribonucleotide with antithrombotic, anti-ischemic, and thrombolytic effects. Defibrotide stimulates fibrinolysis by increasing tissue factor pathway inhibitor and t-PA levels and by decreasing thrombin generation and plasminogen activator inhibitor-1 levels. Notably, defibrotide has no anticoagulant activity, which decreases the possibility of life-threatening hemorrhage. Richardson et al were the first to report the outcomes of defibrotide treatment in 19 patients with VOD after SCT.^[10]^ Their study showed complete resolution of VOD in 8 (42%) patients without severe bleeding. Chopra et al reported similar results of defibrotide therapy in 40 patients, with a 55% complete response rate and 43% survival rate over 100 days.^[26]^

To the best of our knowledge, there are only 2 case reports on defibrotide treatment for patients with VOD after LT. Mor et al reported 2 cases of VOD after LT, treated with defibrotide.^[11]^ In their study, the first patient showed complete clinical resolution of VOD, but the second patient, treated at a later stage of VOD, died 2 months after multiorgan failure due to sepsis. Senzolo et al also reported performing defibrotide treatment for 2 patients with VOD after LT; both patients had severe VOD and showed no improvement after defibrotide administration.^[27]^ These 2 studies have suggested that the role of defibrotide treatment may be optimized with early intervention and restricted to the early stage of VOD after LT. In the present case, serum AST and ALT levels gradually decreased after conservative therapy, especially after switching the immunosuppressive agent from tacrolimus to cyclosporine, serum bilirubin remained at a high level, and the patient's symptoms such as jaundice, ascites, weight gain, and abdominal pain did not improve. After prompt administration of defibrotide on POD 23, serum bilirubin levels dramatically decreased, and the patient's symptoms were alleviated.

In conclusion, defibrotide treatment in this patient with VOD after LDLT led to full recovery. Additionally, this is the first case report of successful defibrotide treatment of VOD after LDLT. Although VOD is a rare complication after LT, its clinical features should be considered for early diagnosis, early intervention, and improved outcomes. Defibrotide is a promising drug for the treatment of VOD after LT, and further large-scale, prospective studies of defibrotide for VOD in patients who underwent LT are required.

## Author contributions

**Conceptualization:** Kwangho Yang.

**Data curation:** Hyo Jung Ko.

**Formal analysis:** Byung Hyun Choi.

**Funding acquisition:** Kwangho Yang.

**Investigation:** Jae Ryong Shim.

**Project administration:** Je Ho Ryu.

**Supervision:** Je Ho Ryu.

**Validation:** Byung Hyun Choi.

**Visualization:** Jung Hee Lee.

**Writing – original draft:** Tae Beom Lee.

**Writing – review & editing:** Tae Beom Lee.

## References

[1] Wadleigh M Ho V Momtaz P Richardson P. Hepatic veno-occlusive disease: pathogenesis, diagnosis and treatment. Curr Opin Hematol 2003;10:451–62. 14564177 10.1097/00062752-200311000-00010

[2] Bras G Jellife DB Stuart KL. Veno-occlusive disease of liver with nonportal type of cirrhosis, occurring in Jamaica. AMA Arch Pathol 1954;57:285–300. 13147641

[3] Dignan FL Wynn RF Hadzic N. BCSH/BSBMT guideline: diagnosis and management of veno-occlusive disease (sinusoidal obstruction syndrome) following haematopoietic stem cell transplantation. Br J Haematol 2013;163:444–57. 24102514 10.1111/bjh.12558

[4] Sebagh M Debette M Samuel D. “Silent” presentation of veno-occlusive disease after liver transplantation as part of the process of cellular rejection with endothelial predilection. Hepatology 1999;30:1144–50. 10534334 10.1002/hep.510300514

[5] Sebagh M Azoulay D Roche B. Significance of isolated hepatic veno-occlusive disease/sinusoidal obstruction syndrome after liver transplantation. Liver Transpl 2011;17:798–808. 21351239 10.1002/lt.22282

[6] Dhillon AP Burroughs AK Hudson M Shah N Rolles K Scheuer PJ. Hepatic venular stenosis after orthotopic liver transplantation. Hepatology 1994;19:106–11. 8276346

[7] Yamada N Urahashi T Ihara Y. Veno-occlusive disease/sinusoidal obstruction syndrome associated with potential antibody-mediated rejection after pediatric living donor liver transplantation: a case report. Transplant Proc 2012;44:810–3. 22483502 10.1016/j.transproceed.2012.01.008

[8] Bearman SI Anderson GL Mori M Hinds MS Shulman HM McDonald GB. Venoocclusive disease of the liver: development of a model for predicting fatal outcome after marrow transplantation. J Clin Oncol 1993;11:1729–36. 8355040 10.1200/JCO.1993.11.9.1729

[9] Palmer KJ Goa KL. Defibrotide. A review of its pharmacodynamic and pharmacokinetic properties, and therapeutic use in vascular disorders. Drugs 1993;45:259–94. 7681375 10.2165/00003495-199345020-00007

[10] Richardson PG Elias AD Krishnan A. Treatment of severe veno-occlusive disease with defibrotide: compassionate use results in response without significant toxicity in a high-risk population. Blood 1998;92:737–44. 9680339

[11] Mor E Pappo O Bar-Nathan N. Defibrotide for the treatment of veno-occlusive disease after liver transplantation. Transplantation 2001;72:1237–40. 11602848 10.1097/00007890-200110150-00009

[12] Shulman HM Gown AM Nugent DJ. Hepatic veno-occlusive disease after bone marrow transplantation. Immunohistochemical identification of the material within occluded central venules. Am J Pathol 1987;127:549–58. 2438942 PMC1899766

[13] Zhou H Wang YX Lou HY Xu XJ Zhang MM. Hepatic sinusoidal obstruction syndrome caused by herbal medicine: CT and MRI features. Korean J Radiol 2014;15:218–25. 24643319 10.3348/kjr.2014.15.2.218 PMC3955788

[14] Yang S Wu J Lei S. CT features of hepatic veno-occlusive disease: a meta-analysis. Acad Radiol 2018;25:328–37. 29191686 10.1016/j.acra.2017.10.012

[15] Takamura H Nakanuma S Hayashi H. Severe veno-occlusive disease/sinusoidal obstruction syndrome after deceased-donor and living-donor liver transplantation. Transplant Proc 2014;46:3523–35. 25498084 10.1016/j.transproceed.2014.09.110

[16] Sanei MH Schiano TD Sempoux C Fan C Fiel MI. Acute cellular rejection resulting in sinusoidal obstruction syndrome and ascites postliver transplantation. Transplantation 2011;92:1152–8. 21993182 10.1097/TP.0b013e318234119d

[17] Shen T Feng XW Geng L Zheng SS. Reversible sinusoidal obstruction syndrome associated with tacrolimus following liver transplantation. World J Gastroenterol 2015;21:6422–6. 26034381 10.3748/wjg.v21.i20.6422 PMC4445123

[18] Shin SH Yahng SA Yoon JH Lee SE Cho BS Kim YJ. Hepatic veno-occlusive disease resulting in tacrolimus toxicity after allogeneic hematopoietic stem cell transplantation. Blood Res 2013;48:55–7. 23589797 10.5045/br.2013.48.1.55 PMC3625009

[19] Shen T Tang X Xiang H Zheng S. Graft failure from hepatic veno-occlusive disease after a liver transplant: a case report. Exp Clin Transplant 2016;14:575–9. 25365427 10.6002/ect.2014.0182

[20] DeLeve LD Valla DC Garcia-Tsao G. American Association for the Study Liver Diseases. Vascular disorders of the liver. Hepatology 2009;49:1729–64. 19399912 10.1002/hep.22772 PMC6697263

[21] Bearman SI Lee JL Barón AE McDonald GB. Treatment of hepatic venoocclusive disease with recombinant human tissue plasminogen activator and heparin in 42 marrow transplant patients. Blood 1997;89:1501–6. 9057629

[22] Richardson PG Ho VT Cutler C Glotzbecker B Antin JH Soiffer R. Hepatic veno-occlusive disease after hematopoietic stem cell transplantation: novel insights to pathogenesis, current status of treatment, and future directions. Biol Blood Marrow Transplant 2013;19:S88–90. 23089567 10.1016/j.bbmt.2012.10.023

[23] Haire WD Ruby EI Gordon BG. Multiple organ dysfunction syndrome in bone marrow transplantation. JAMA 1995;274:1289–95. 7563534

[24] Ibrahim A Pico JI Maraninchi D. Hepatic veno-occlusive disease following bone marrow transplantation treated by prostaglandin E1. Bone Marrow Transplant 1991;7: Suppl 2: 53. 1878718

[25] Senzolo M Cholongitas E Patch D Burroughs AK. TIPS for venoocclusive disease: is the contraindication real? Hepatology 2005;42:240–1. 15962296 10.1002/hep.20773

[26] Chopra R Eaton JD Grassi A. Defibrotide for the treatment of hepatic veno-occlusive disease: results of the European compassionate-use study. Br J Haematol 2000;111:1122–9. 11167751 10.1046/j.1365-2141.2000.02475.x

[27] Senzolo M Patch D Cholongitas E. Severe venoocclusive disease after liver transplantation treated with transjugular intrahepatic portosystemic shunt. Transplantation 2006;82:132–5. 16861953 10.1097/01.tp.0000225799.76828.ce

